# Measles Outbreak in South Africa: Epidemiology of Laboratory-Confirmed Measles Cases and Assessment of Intervention, 2009–2011

**DOI:** 10.1371/journal.pone.0055682

**Published:** 2013-02-20

**Authors:** Genevie M. Ntshoe, Johanna M. McAnerney, Brett N. Archer, Sheilagh B. Smit, Bernice N. Harris, Stefano Tempia, Mirriam Mashele, Beverley Singh, Juno Thomas, Ayanda Cengimbo, Lucille H. Blumberg, Adrian Puren, Jocelyn Moyes, Johann van den Heever, Barry D. Schoub, Cheryl Cohen

**Affiliations:** 1 Centre for Vaccines and Immunology, National Institute for Communicable Diseases (NICD) of the National Health Laboratory Service (NHLS), Johannesburg, South Africa; 2 Centre for Respiratory Diseases and Meningitis, National Institute for Communicable Diseases of the National Health Laboratory Service, Johannesburg, South Africa; 3 Division of Public Health Surveillance and Response, National Institute for Communicable Diseases of the National Health Laboratory Service, Johannesburg, South Africa; 4 School of Health Systems and Public Health, University of Pretoria, Pretoria, South Africa; 5 Influenza Division, United States Centers for Disease Control and Prevention (CDC), Attaché to the NICD-NHLS, Johannesburg, South Africa; 6 Centre for HIV and STI, National Institute for Communicable Diseases of the National Health Laboratory Service, Johannesburg, South Africa; 7 Expanded Programme on Immunisation in South Africa (EPI SA), Department of Health, Pretoria, South Africa; University of Missouri-Columbia, United States of America

## Abstract

**Background:**

Since 1995, measles vaccination at nine and 18 months has been routine in South Africa; however, coverage seldom reached >95%. We describe the epidemiology of laboratory-confirmed measles case-patients and assess the impact of the nationwide mass vaccination campaign during the 2009 to 2011 measles outbreak in South Africa.

**Methods:**

Serum specimens collected from patients with suspected-measles were tested for measles-specific IgM antibodies using an enzyme-linked immunosorbent assay and genotypes of a subset were determined. To estimate the impact of the nationwide mass vaccination campaign, we compared incidence in the seven months pre- (1 September 2009–11 April 2010) and seven months post-vaccination campaign (24 May 2010–31 December 2010) periods in seven provinces of South Africa.

**Results:**

A total of 18,431 laboratory-confirmed measles case-patients were reported from all nine provinces of South Africa (cumulative incidence 37 per 100,000 population). The highest cumulative incidence per 100,000 population was in children aged <1 year (603), distributed as follows: <6 months (302/100,000), 6 to 8 months (1083/100,000) and 9 to 11 months (724/100,000). Forty eight percent of case-patients were ≥5 years (cumulative incidence 54/100,000). Cumulative incidence decreased with increasing age to 2/100,000 in persons ≥40 years. A single strain of measles virus (genotype B3) circulated throughout the outbreak. Prior to the vaccination campaign, cumulative incidence in the targeted vs. non-targeted age group was 5.9-fold higher, decreasing to 1.7 fold following the campaign (P<0.001) and an estimated 1,380 laboratory-confirmed measles case-patients were prevented.

**Conclusion:**

We observed a reduction in measles incidence following the nationwide mass vaccination campaign even though it was conducted approximately one year after the outbreak started. A booster dose at school entry may be of value given the high incidence in persons >5 years.

## Introduction

Measles is a highly infectious disease and may cause extensive epidemics [1]–[2]. Despite the availability of a safe and highly effective vaccine, measles still remains one of the leading causes of vaccine-preventable deaths in children <5 years of age worldwide, especially in developing countries, with up to 20% of these deaths occurring in those <1 year [2]–[4]. In the 1990s it was estimated that about 45 million cases and one million measles deaths occurred worldwide [5]. However in 2008 the number decreased to an approximated 20 million or more cases and 164,000 deaths with over 95% of these occurring in low-income countries with poor health systems [4].

In the World Health Organisation (WHO) African region, routine measles vaccination is offered at nine months of age but about 15% of children vaccinated at this age will not develop protective immune response [6]. In addition, not all children will receive measles vaccine. As a result the number of susceptible individuals may accumulate over time with the potential for outbreaks to occur. To prevent this, a second opportunity for measles vaccination is offered through routine services or supplemental immunization activities (SIAs). However, to eliminate measles, coverage for both routine schedule and SIAs must be maintained at >95% throughout the country [7]. The WHO guidelines for response to measles outbreaks have been recently updated to include recommendations for non-selective vaccination campaigns in certain settings [8]. Other countries in Africa have conducted outbreak-response vaccination during measles outbreaks and have showed potential benefits during prolonged measles outbreaks [9]–[10].

In South Africa, routine measles vaccination at nine months of age was introduced in 1975; and a second routine vaccination dose at 18 months of age was added in 1995 [11]. In addition, supplemental vaccination campaigns were conducted every four years between 1996 and 2004 and then every 3 years since 2004 (due to suboptimal routine coverage, high drop-out rate between the 1^st^ and 2^nd^ doses and suboptimal campaign coverage in 2004), with coverage ranging from 77% to 93% [11]–[13]. A target was set by the South African health authorities to eliminate indigenous measles transmission by the year 2002 [11]. To achieve this, South Africa adopted and implemented the measles elimination strategies defined by the WHO [14]. However, a large measles outbreak occurred between July 2003 and November 2005 involving 1,676 laboratory-confirmed case-patients in five provinces with sporadic cases in four other provinces of South Africa [11]. According to the manuscript authors, the likely cause of this outbreak was failure to achieve adequate vaccination coverage [11]. A periodic nationwide supplementary measles vaccination campaign targeting children aged nine months to four years was conducted in July 2004, achieving a national coverage of 92% [Personal communication National Department of Health (NDoH)]. Following this outbreak, South Africa reported relatively low annual numbers of measles IgM positive case-patients ranging from 32 to 82 through the years 2006 to 2008 [15]–[17]. A second large measles outbreak occurred in 2009 to 2011.

There is a dearth of information about the possible causes of recurrent measles outbreaks and little data on the effectiveness of outbreak-response vaccination in African countries [9]–[10]. We analyzed the epidemiological characteristics of the 2009 to 2011 measles outbreak and discuss the findings in relation to the measles control practices in the country. In addition, we evaluated the effectiveness of the 2010 nationwide periodic supplementary measles vaccination campaign in seven of the nine provinces in South Africa.

## Methods

### Data source

Data presented here represent measles patients from whom laboratory results were received during the 2009–2011 measles outbreak by the National Institute for Communicable Diseases (NICD) of the National Health Laboratory Service (NHLS) from 16 March 2009 to 31 August 2011 (study period). Data included the following variables: demographic characteristics (age and sex); location (name of health facility, province and district) and time (date specimens were collected, received and tested). Date of rash onset, vaccination history, severity and outcome data were not available as they are often not submitted to the laboratory. Administrative vaccine coverage for the nationwide mass measles vaccination campaign conducted in 2010 (12 April to 9 May), was obtained from the NDoH, South Africa. The 2010 mid-year population estimates used for the cumulative incidence calculations were acquired from Statistics South Africa [18].

### Sample collection and laboratory testing

In South Africa measles is a notifiable disease and all patients meeting the suspected-measles case definition (rash and fever with at least one of cough, coryza or conjunctivitis) should have specimens taken. Blood and throat/nasopharyngeal swab or urine specimens are sent on ice to the NICD-NHLS for laboratory confirmation where testing is conducted at no charge. In addition, specimens from all patients diagnosed with measles by private sector laboratories are referred to the NICD-NHLS laboratory for confirmatory testing. The recommendation for laboratory confirmation of all suspected-measles cases nationally remained throughout the measles outbreak; however, the Western Cape Province suspended routine laboratory confirmation from week starting 8 March (week 10) to week ending 30 May (week 21) 2010.

Serum specimens were tested for the presence of measles-specific immunoglobin (IgM) antibodies using an enzyme-linked immunosorbent assay (ELISA), (Enzygnost, Siemens, Marburg, Germany). A geographically and temporally representative subset of specimens from individuals of all ages with reactive measles IgM serology results were tested for molecular characterization by reverse transcriptase polymerase chain reaction (RT-PCR) and nested PCR. Subset of specimens were sampled in the beginning of the outbreak, towards the end of the year 2009, beginning of and mid 2010 and towards the end of the outbreak and were from different geographic areas in all nine provinces. RNA was extracted directly from clinical specimens (blood or urine) and tested for the presence of measles virus. The genotype was determined through sequencing of the amplicons and phylogenetic analysis of the viral nucleoprotein (N) gene.

### Data management and analysis

Data were analysed using OpenEpi version 2.3.1 and Epi Info version 3.5.3 (US Centers for Disease Control and Prevention, Atlanta, Georgia). A p-value of <0.05 was considered to be statistically significant. Measles cumulative incidence (pre- and post-campaign periods) were calculated by dividing the number of laboratory-confirmed cases by the population at risk over a specified time period.

To estimate the impact of the nationwide vaccination campaign, we calculated the following for the 7 months pre- and 7 months post-vaccination campaign periods in seven of the nine provinces: cumulative measles incidence and incidence ratios among the targeted and non-targeted age groups. The period of 7 months was chosen as this was the period from the start of the national outbreak until the vaccination campaign. We made assumptions as described by Goodson *et* *al* [10] that (1) if nationwide vaccination campaign was not conducted, age distribution would be similar for the duration of the outbreak and as a result the cumulative incidence ratio would remain constant and (2) the nationwide vaccination campaign did not affect transmission in the non-targeted age group. We calculated the expected cumulative incidence in the absence of the nationwide vaccination campaign using the formula described by Goodson *et* *al* [10]. Subsequently the expected cumulative incidence was used to approximate the number of cases prevented by the vaccination campaign.

Gauteng Province was excluded from the assessment as outbreak-response vaccination campaigns were conducted in 2009. Western Cape Province was also excluded as routine measles testing was stopped for a period of 12 weeks surrounding the nationwide vaccination campaign. For the remaining seven provinces, measles case-patients were divided into three groups according to the date specimens were collected:

- Pre-campaign group – specimens collected up to seven months before the campaign period 1 September 2009 to 11 April 2010 (N = 7,084).
- During campaign group – specimens collected during the four week campaign period (12 April to 9 May 2010) plus two additional weeks (10 to 23 May 2010) to allow for the development of an immune response (N = 2,163).
- Post-campaign group – specimens collected up to seven months after the campaign was completed 24 May 2010 to 31 December 2010 (N = 1,371).

## Results

### Epidemiological characteristics of patients (all nine provinces)

**Figure 1:**
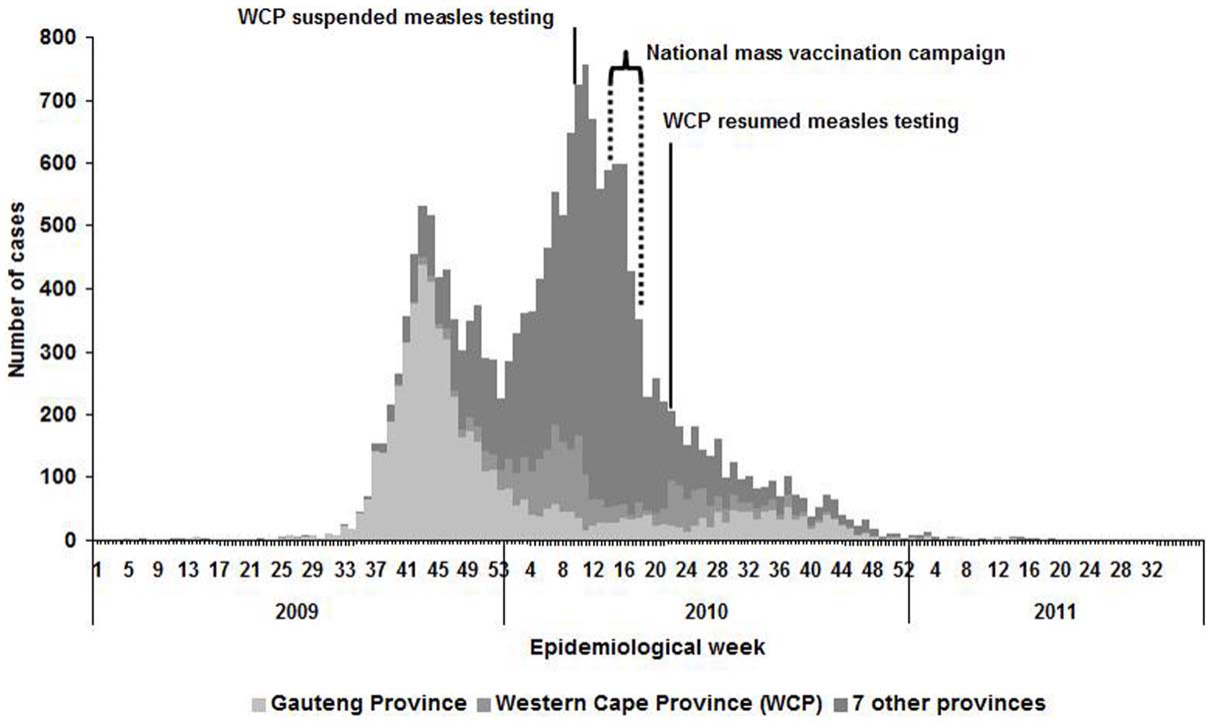
Weekly* number of measles IgM positive cases: South Africa, 2009–2011. * Week calculated from date of specimen collection (n = 17 351), date received (n = 1 067) or date tested (n = 13).

**Figure 2:**
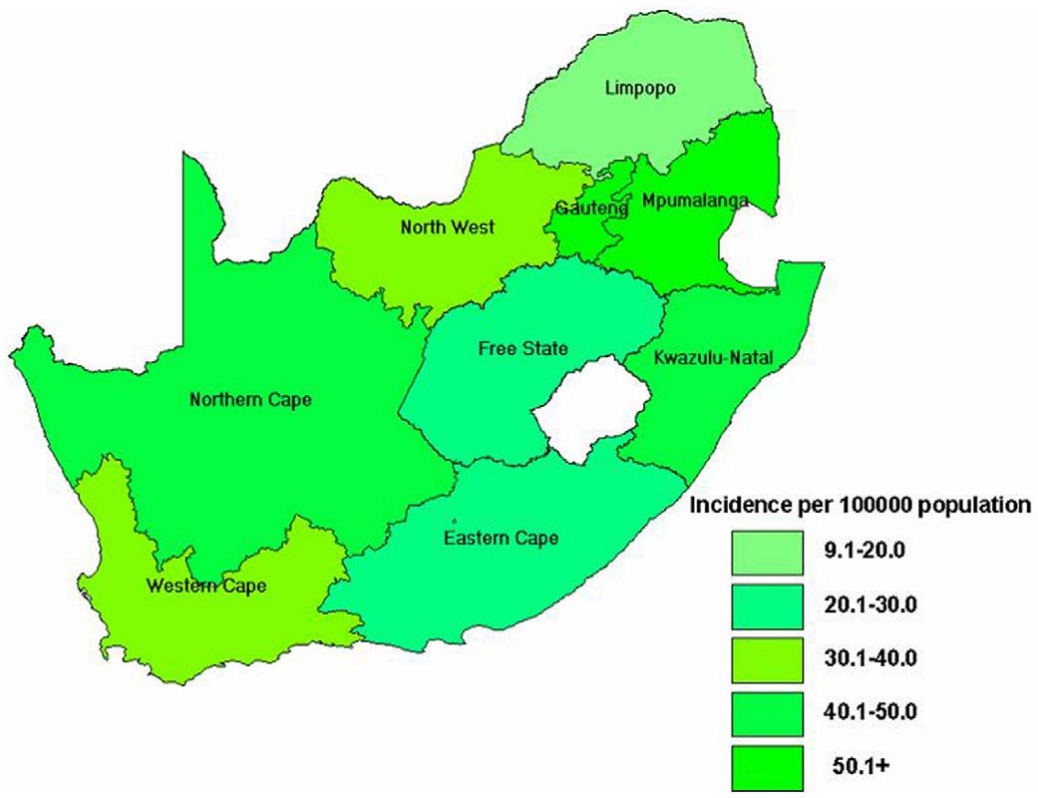
Map showing nine South African provinces and overall cumulative incidence per 100 000 population by province, 2009–2011.

For the period 1 January 2009 to 31 August 2011, the NICD-NHLS tested about 45,452 specimens that were collected from suspected measles cases nationally. From 1 January to 15 March 2009, 10 sporadic laboratory-confirmed measles case-patients were reported from five of the nine provinces. The first laboratory-confirmed measles case-patient related to this outbreak was identified from a specimen collected on 16 March 2009 (week 10) from Gauteng Province. A marked increase in the number of laboratory-confirmed measles cases was noted from epidemiologic week 25, 2009 (week ending 21 June) onward, with a primary peak occurring in October 2009 (week 43) and a secondary peak in March 2010 (week 11) before gradually declining to relatively low numbers at the end of the year 2010 (Figure 1). From 16 March 2009 to 31 August 2011, there were a total of 18,431 laboratory-confirmed measles case-patients nationally, with a cumulative incidence of 37 per 100,000 population. Case-patients were reported from all nine provinces, with Gauteng (31%, 5,762/18,431), KwaZulu-Natal (23%, 4,283/18,431) and Western Cape (11%, 2,009/18,431) provinces accounting for the largest proportions of the total. Cumulative incidence differed by province with Mpumalanga and Gauteng provinces being the most affected (Figure 2).

**Figure 3:**
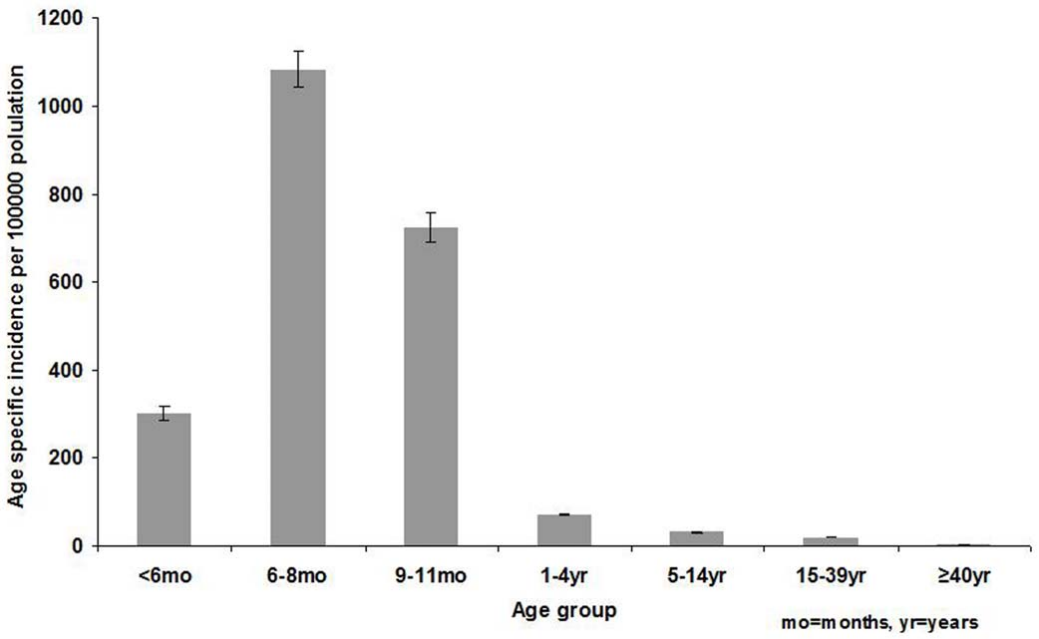
Age-specific incidence of laboratory-confirmed measles cases, South Africa, 2009–2011.

Age and sex were known for 95% (17,530/18,431) and 96% (17,763/18,431) of laboratory-confirmed measles case-patients respectively. Children <5 years accounted for 52% (9,035/17,530) of cases, with 35% (6,122/17,530) aged <1 year. Of those aged <1 year, 70% (4,284/6,122) were aged <9 months. Forty eight percent of case-patients were ≥5 years with a cumulative incidence of 54/100,000. The highest cumulative incidence was in children aged <1 year (603/100,000 population), distributed as follows: <6 months (302/100,000), 6 to 8 months (1083/100,000) and 9 to 11 months (724/100,000). Cumulative incidence decreased with increasing age to low levels (2/100,000) in persons ≥40 years (Figure 3).

### Genotypes

Of 425 specimens tested by PCR, 197 were PCR positive; 193 were outbreak-related wild-type measles virus (genotype B3) while the remaining two were travel-related and were identified as imported wild-type virus (genotype D8 from India and genotype D4 from France) while two were vaccine related.

### Supplementary vaccination coverage

A planned nationwide periodic supplementary vaccination campaign was conducted in eight of the nine provinces in 2010 (12 April to 9 May), with a “mop-up” campaign in Gauteng Province. Supplementary vaccination campaigns are performed every 3 to 4 years, depending on routine vaccination coverage and previous campaign coverage – the lower the routine and previous campaign coverage, the shorter the inter-campaign period. The periodic campaigns in the past targeted children aged 9 to 59 months; however the 2010 campaign was expanded to include children aged 6 months to 14 years regardless of their vaccination status. South Africa achieved an overall administrative coverage of 90.5% in eight of the nine provinces. An estimated coverage of >100% was achieved for those aged 6 months to 4 years and 84% for those aged 5 to 14 years (Table 1).

**Table 1:** Reported administrative vaccination coverage* by province during the nationwide mass vaccination campaign, South Africa, May/April 2010.

Province	Population estimates		Coverage in %		
	6 mo to 4 yr	5 to 14 yr	6 mo to 4 yr	5 to 14 yr	Overall
Eastern Cape	645,469	1,469,246	104	80	87
Free State	270,865	597,434	90	79	83
KwaZulu-Natal	1,027,303	2,364,930	113	83	92
Limpopo	511,447	1,259,993	118	90	98
Mpumalanga	344,320	833,713	124	91	100
Northern Cape	103,743	252,249	108	90	95
North West	321,039	720,433	102	79	86
Western Cape	452,738	1,020,130	99	76	83
**South Africa** **	**3,676,921**	**8,518,128**	**107**	**84**	**91**

* Administrative method: number of doses delivered divided by the target population which is determined from the census projections.

mo = months; yr = years.

** 8 of 9 provinces of South Africa included, Gauteng Province excluded as widespread vaccination was conducted in this province in 2009.

Source: Personal Communication: National Department of Health, South Africa.

### Assessment of the 2010 vaccination campaign (Seven of nine provinces)

#### Age-specific measles incidence

Prior to the nationwide vaccination campaign, South Africa (7/9 provinces) reported a cumulative incidence of 20.0 measles cases per 100,000 population, which decreased to 3.8 per 100,000 population in the post-campaign period. The highest cumulative incidence was in children aged <1 year, with a cumulative incidence of 284 per 100,000 population, distributed as follows: <6 months (121/100,000), 6 to 8 months (487/100,000), and 9 to 11 months (407/100,000). Cumulative incidence decreased with increasing age to low levels in those aged ≥40 years (0.6 per 100,000 population). In the post-campaign period, a reduction in measles incidence was observed in all age groups (Table 2).

**Table 2:** Characteristics of laboratory-confirmed measles cases before and after the vaccination campaign in seven South African provinces, 2009–2010.

	Pre-campaign* 1 September 2009–11 April 2010 (N = 7 084)		Post-campaign^#^ 24 May 2010–31 December 2010 (N = 1 371)			
Characteristics	n (%)	Cumulative Incidence/100 000 population (95% CI)	n (%)	Cumulative Incidence/100 000 population (95% CI)	% Reduction (95% CI)	Rate ratio (95% CI)
**Age group in years**						
<1yr	2,004 (30)	284.0 (271.7–296.7)	513 (40)	72.7 (66.5–79.3)	74 (71.8–76.8)	0.26 (0.23-0.28)
<6mo	426	120.7 (109.5–132.8)	170	48.2 (41.2–56.0)	60 (52.3–66.6)	0.40 (0.33-0.48)
6–8mo	860	487.5 (455.4–521.2)	244	138.3 (121.5–156.8)	72 (67.3–75.4)	0.28 (0.25-0.33)
9–11mo	718	407.0 (377.8–437.9)	99	56.1 (45.6–68.3)	86 (83.0–88.8)	0.14 (0.11-0.17)
1–4yr	1,372 (20)	48.2 (45.7–50.9)	140 (11)	4.9 (4.1–5.8)	90 (87.9–91.4)	0.10 (0.08-0.12)
5–14yr	1,975 (29)	26.6 (25.4–27.8)	92 (7)	1.2 (1.0–1.5)	95 (94.3–96.2)	0.05 (0.04-0.06)
15–39yr	1,348 (20)	7.7 (7.3–8.1)	522 (40)	3.0 (2.7–3.2)	61 (57.2–65.0)	0.39 (0.35-0.43)
> = 40yr	30 (0.4)	0.6 (0.4–0.8)	24 (2)	0.5 (0.3–0.7)	20 (−36.8–53.2)	0.80 (0.47-1.37)
All ages	7,084	21.1 (20.6–21.6)	1,371	4.1 (3.9–4.3)	81 (79.5–81.7)	0.19 (0.18-0.20)
**Sex**						
Male	3,491 (51)	21.5 (20.8–22.2)	745 (53)	4.6 (4.3–4.9)	79 (76.9–80.3)	0.21 (0.20-0.23)
Female	3,375 (49)	19.4 (18.8–20.1)	574 (47)	3.3 (3.0–3.6)	83 (81.4–84.4)	0.17 (0.15-0.18)
**Province**						
Eastern Cape	1,058 (15)	15.7 (14.8–16.70	106 (8)	1.6 (1.3–1.9)	90 (87.8–91.8)	0.10 (0.08-0.12)
Free State	453 (6)	16.0 (14.6–17.6)	237 (17)	8.4 (7.4–9.5)	48 (38.8–55.3)	0.52 (0.45-0.61)
KwaZulu-Natal	2,747 (39)	25.8 (24.8–26.8)	480 (35)	4.5 (4.1–4.9)	83 (80.8–84.1)	0.17 (0.16-0.19)
Limpopo	408 (6)	7.5 (6.8–8.3)	29 (2)	0.5 (0.3–0.8)	93 (89.6–95.1)	0.07 (0.05-0.10)
Mpumalanga	1,221 (17)	33.8 (31.9–35.7)	263 (19)	7.3 (6.4–8.2)	78 (75.4–81.1)	0.21 (0.19-0.25)
Northern Cape	229 (3)	20.7 (18.1–23.6)	141 (10)	12.8 (10.7–15.1)	38 (24.1–50.1)	0.62 (0.50-0.76)
North West	968 (14)	30.2 (28.4–32.2)	115 (8)	3.6 (3.0–4.3)	88 (85.6–90.2)	0.12 (0.10-0.14)
**Group**						
Targeted	4,925 (73)	46.4 (45.1–47.7)	575 (45)	5.4 (5.0–5.9)	88 (87.3–89.3)	0.12 (0.11-0.13)
Non-targeted	1,804 (26)	7.9 (7.5–8.2)	716 (55)	3.1 (2.9–3.4)	60 (56.7–63.6)	0.40 (0.36-0.43)

* Age and sex known in 6,729 and 6,866 cases respectively; **#** Age and sex known in 1,291 and 1,319 cases respectively; mo = months; yr = years.

Please note: population for the <1 year unavailable per month; as a result, the population was estimated by dividing the population of <1 year by 12.

#### Provincial measles incidence

The highest cumulative incidence in the pre-campaign period was observed in Mpumalanga Province (33.8 per 100,000 population) while the lowest was observed in Limpopo (7.5 per 100,000). Following the nationwide vaccination campaign a reduction in cumulative incidence was observed in all provinces (Table 2).

#### Estimated effect of the vaccination campaign

Prior to the nationwide vaccination campaign cumulative incidence among the target age group was 46.4 per 100,000 population, which decreased to 5.4 per 100,000 post-campaign period. In the pre-campaign period, the cumulative incidence amongst the target age group was 5.9 fold higher than in the non-targeted age group (46.4 vs. 7.9 per 100,000 population). In the post-campaign period the cumulative incidence amongst the target age group was 1.7 fold higher than the non-target group (5.4 vs. 3.1 per 100,000 population) (Table 2). The expected cumulative incidence (assuming no vaccination intervention) among the target age group was found to be 18.3 per 100,000 population, 3.4 fold higher than the observed 5.4 per 100,000. As a result an estimated 1,380 laboratory-confirmed measles case-patients were prevented in seven of nine provinces assessed.

## Discussion

We described a widespread measles outbreak involving 18,431 laboratory-confirmed measles case-patients, the largest outbreak since 1992 when more than 22,000 clinically diagnosed case-patients were reported [14]. Due to challenges with submission and collation of clinical notifications, laboratory confirmation was conducted throughout the course of the outbreak, even though this is not a WHO recommendation [8]. As our analysis was restricted to laboratory-confirmed case-patients it represents a minimum estimate of the total number of persons infected. Children aged <1 year were the most affected age group especially those <9 months. Even though the nationwide periodic vaccination campaign was conducted late in the outbreak course (13 months after the first case-patients were reported), we estimate that over 1,300 laboratory-confirmed case-patients were prevented.

Similar to the measles outbreak from 2003 through 2005, the 2009 to 2011 outbreak began in Gauteng Province [11]. Possible reasons for this include the fact that Gauteng Province has a high density population and experiences relatively high rates immigration from other areas and countries [18]. The numbers of reported case-patients differed between provinces. Variation in reported case-numbers by geographic area may be affected by differential access to care between urban and rural areas, as well as differences in laboratory specimen taking practices and underreporting in some areas.

A high proportion of cases (24%) were aged <9 months, those not eligible to receive the first dose of measles vaccine from routine immunisation in South Africa. Our data indicate that there is a substantial immunity gap in this group, possibly due to waning maternal antibodies in the setting when immunity is from vaccination not natural infection, [19]–[20] compounded by HIV exposure [21]–[24]. Disease in this age group is of concern as they are at high risk for severe and complicated measles. By 6 months of age, HIV-1 infected infants have lower antibody levels that are unlikely to affect immune response to measles vaccine [24]. Lepage *et* *al* demonstrated a higher seroconversion rates in HIV-infected children vaccinated at 6 months [25]. In South Africa, a supplemental dose of measles vaccine at six months of age currently is recommended for infants at high risk especially HIV-infected, HIV-exposed [Unpublished: Integrated management of childhood illness, IMCI, South Africa 2010, HIV infection in children, Module S1. Draft version] and infants admitted to hospitals [26]; however implementation of this recommendation is variable.

Although the highest cumulative incidence was in those aged <5 years, 48% were reported in those aged ≥5 years. This suggests that a significant immunity gap existed in this age group, likely due to the accumulation of susceptible individuals over several years. An additional booster dose at school entry, which is currently not part of the routine schedule in South Africa, would be of value. Recent outbreaks in Europe have shown a higher proportion (64%) of cases were among patients aged ≥5 years [27]; however, in France the highest incidence was observed in those aged <1 year [28].

In 2010, South Africa conducted a non-selective nationwide vaccination campaign that showed an impact, even though it was conducted late in the outbreak course. A reduction in incidence of laboratory-confirmed measles case-patients was observed in all age groups following the nationwide vaccination campaign with a shift in the age distribution of cases away from the targeted age group similar to what has been described in other African settings [10]. However, cases continued to be reported within this age group indicating that despite the vaccination campaign an immunity gap continued to exist in this group. More cases could have been averted if the campaign was carried out earlier. It is known that outbreak-response vaccination may fail to contain spread if not conducted promptly, within a short space of time to a wide age range or if the coverage is not adequately high enough [8], [29]. The campaign could not be brought forward due to challenges in procuring adequate volumes of registered vaccines and effectively organizing a campaign of such magnitude within a short space of time. Even given these limitations, it does appear that the campaign did have an impact in reducing the total number of measles case-patients and possibly also shortened the duration of the outbreak.

A single outbreak strain was detected, which is reflective of an outbreak with a low vaccine coverage setting. This particular strain of B3 has not circulated in South Africa previously. Genotype B3 viruses have been identified as circulating endemically in West and Central Africa [30]. It is thus likely that this strain of genotype B3 was imported into South Africa from this region. During 2009 to 2010 other African countries also experienced measles outbreaks; these were predominantly caused by strains of genotype B3 [31].

Our study had several limitations. First, data presented here relate only to laboratory-confirmed measles case-patients tested at the NICD-NHLS and do not include case-patients where no specimens were taken or from specimens that were tested only at the private laboratories. Second, we may have underestimated the number of cases as testing efficiencies may be greater in better resourced provinces and not all patients will seek medical advice. In addition clinicians may be more likely to test children. Third, we could not comment on the clinical presentation and/or severity of cases, vaccination status and mortality data as these information is rarely submitted to the laboratory. Fourth, our estimates of vaccination coverage were based on administrative data, which depend on the validity of the numerators or denominators and therefore can only provide a relatively imprecise estimation of the vaccination coverage. Reported coverage figures of >100% in some areas are likely a result of use of denominators extrapolated from the 2001 census. Unfortunately no post campaign surveys were conducted. Lastly, we may have underestimated the impact of the vaccination campaign as cases prevented during the campaign period were excluded from the analysis as it is expected to see the impact from as early as 72 hrs to the first 2 weeks of campaign. In addition, unvaccinated persons may have benefited from the indirect effect of vaccination in the target age group and the two provinces with the highest numbers of cases were excluded from the analysis. In addition the campaign in Gauteng could have reduced case numbers in neighbouring provinces [10]. Numbers of reported cases were decreasing in some provinces at the time the campaign was conducted, thus some of the reductions could have been due to the epidemic burning out. We did however; attempt to adjust for this by controlling for trends in the age groups not targeted for vaccination. The contribution of seasonal forces to the outbreak is unclear although peaks in incidence in the outbreak were observed in Autumn and Spring, the peak seasonal periods prior to widespread introduction of measles vaccine in South Africa was also experienced during Spring and Autumn each year [14]. Apart from reduced testing in the Western Cape Provinces there were no documented changes in reporting efficiency following the campaign.

In conclusion, this nationwide outbreak, affecting a wide age range, highlights that South Africa remains vulnerable to large measles outbreaks. Efforts to maintain high routine measles vaccination coverage should be emphasized. These can be strengthened by conducting biannual analysis of the potential risk of measles outbreaks with action plans to improve routine vaccination coverage if below 90%. Specific interventions such as immunisation awareness days can also be held to reach unvaccinated children and/or those who did not develop protective immune responses; thus lessening the number of susceptible individuals. Furthermore, efforts to conform to the recommendations for a measles vaccine dose at six months for infants at high risk should be strengthened. In addition, a booster dose at school entry should be considered. Our findings indicate that mass measles vaccination campaigns conducted during the course of an outbreak may reduce total case numbers affected in countries where there is a potential for a large outbreak to occur. However, the decision to conduct a campaign should be based on a comprehensive risk-benefit assessment including assessment of the potential for epidemic spread and the costs of the intervention.
